# Stroke Risk Associated With Lowering Elevated Blood Pressure in Patients With Transient Ischemic Attack

**DOI:** 10.7759/cureus.78736

**Published:** 2025-02-08

**Authors:** Alexsandra Biel, Jacob Keeley, Brett Todd

**Affiliations:** 1 Emergency Medicine, Oakland University William Beaumont School of Medicine, Rochester, USA; 2 Biostatistics, Oakland University William Beaumont School of Medicine, Rochester, USA; 3 Emergency Medicine, Corewell Health William Beaumont University Hospital, Royal Oak, USA

**Keywords:** antihypertensive therapy, emergency department, hypertension, stroke, transient ischemic attack

## Abstract

Background

Hypertension is a risk factor for developing stroke after transient ischemic attack (TIA), yet it is unknown if stroke risk is altered by emergency department (ED) antihypertensive therapy. We aimed to investigate stroke rate in a population of TIA patients presenting with elevated blood pressure in the ED, comparing those who received antihypertensive medication in the ED to those who received no treatment. Secondarily, we aimed to assess the association between ED antihypertensive therapy and intensive care unit (ICU) admit rates, hospital length of stay (LOS), and discharge disposition setting in this population.

Methods

We conducted a retrospective cohort study evaluating adult TIA patients presenting with elevated blood pressure (diastolic ≥ 140 mm Hg or systolic ≥ 90 mm Hg) at any of our Metro Detroit hospital system’s EDs between August 2016 and April 2022. We collected data on age, sex, race, blood pressure in the ED, ED antihypertensive therapy, stroke in the subsequent hospital stay, hospital LOS, ICU admission rates, and discharge disposition. Patient characteristics were summarized using descriptive statistics and two-sample hypothesis testing. We assessed the outcomes of antihypertensive treatment using multivariable logistic regression controlling for patient characteristics.

Results

There were 3,095 patients included in our analysis, of which 21.0% (649) received antihypertensive treatment and 13.9% (429) suffered a stroke. There was no significant difference in stroke rate in the treatment group compared to the no-treatment group (aOR, 1.12 (95% CI, 0.87-1.43)). There was a slightly longer hospital LOS in the treatment group (2.1 days vs 1.9 days), but no differences were seen in ICU admission or discharge disposition.

Conclusion

In TIA patients presenting with elevated blood pressure in the ED, antihypertensive therapy does not appear to be associated with decreased stroke risk in the subsequent hospital stay.

## Introduction

Transient ischemic attack (TIA), a brief neurological deficit caused by an ischemic episode in a focal area of the brain or retina [1,2], presents a significant burden to emergency departments (EDs) in the United States, with at least 240,000 cases yearly and the ED being the location where evaluations most commonly occur [3,4]. There is a strong association between a TIA and the incidence of subsequent stroke and death, and these adverse outcomes typically occur soon after the initial event [5,6]. In fact, prior studies have shown that the risk of developing a stroke within 90 days of a TIA is approximately 10.5% with about half of these occurring in the first one to two days [7]. Risk factors known to increase the likelihood of developing a stroke following a TIA include advanced age, diabetes, TIA duration, symptomatology at the time of presentation, and elevated blood pressure [8-11]. Hypertension, in particular, has been shown to be a major modifiable risk factor in not only the onset of TIA, but also in the subsequent occurrence of stroke [5,12]. Specifically, higher values of both systolic and diastolic blood pressure are linearly associated with a higher incidence of stroke in patients who have had a TIA [5].

A critical element of TIA management is blood pressure control, however, the optimal timing to initiate antihypertensive therapy following a TIA is unclear [13]. As the initial assessment and treatment for TIA typically occurs in the ED, blood pressure management for TIA may be of benefit, however, the effect of antihypertensive medication in TIA patients presenting with a hypertensive level in the ED has not been elucidated. Further, it is unclear how antihypertensive therapy in this patient cohort impacts intensive care unit (ICU) admission rates, hospital length of stay (LOS), and discharge disposition. We aimed to investigate outcomes in TIA patients presenting with elevated blood pressure in ED who received antihypertensive therapy compared to those who did not. We investigated the stroke rate (ischemic and hemorrhagic) following a TIA, as well as hospital LOS, discharge disposition, and ICU admission rate among those receiving antihypertensive therapy in the ED compared to those who did not. As altering BP may reduce the ischemic penumbra in hypertensive TIA patients, we hypothesize that ED antihypertensive therapy in TIA patients presenting with elevated blood pressure is associated with a reduction in subsequent stroke risk without significant effects on hospital LOS, ICU admissions, or discharge disposition.

A research abstract with preliminary data from this study was presented at the American College of Emergency Physicians Research Forum in October 2023.

## Materials and methods

Study design, setting, and participants

We conducted a retrospective observational study of ED patients presenting with TIA and elevated blood pressure. Our primary objective was to determine if there is an association between ED antihypertensive therapy and subsequent decreased stroke risk in this population. Our secondary objectives were to investigate whether ED antihypertensive therapy is associated with an alteration in hospital LOS, discharge disposition setting, and ICU admission in this population. This study was conducted at a large eight-hospital system in Metro Detroit consisting of one tertiary care academic center and seven community hospitals with a diverse range of patients across socioeconomic and ethnic backgrounds. This study was approved by the hospital's institutional review board.

We evaluated all adult patients (greater than or equal to 18 years of age) seen in one of the hospital system's EDs with a diagnosis of TIA (the International Classification of Diseases, 10th Revision (ICD-10) codes G45.9, G45.8) and had a systolic blood pressure 140 mm Hg or greater or a diastolic blood pressure of 90 mm Hg or greater during their ED stay. This blood pressure range is based on guidelines from the Eighth Joint National Committee (JNC8) describing stage 1 hypertension as a systolic blood pressure of between 140 and 159 mm Hg or diastolic blood pressure in the range of 90-99 mm Hg [14,15]. We excluded patients who were pregnant or had pregnancy-related hypertensive diagnoses (i.e., eclampsia or preeclampsia), as well as those who received a thrombolytic agent in the ED as this would be an indicator of stroke (as opposed to a TIA diagnosis) in the ED. Additionally, only those patients with a single isolated TIA visit in the ED were included in the study, as such, those with multiple TIA visits were excluded. This was done to avoid confusion regarding multiple visits for the same TIA.

The study cohort was obtained from the integrated hospital electronic medical record (EMR) system (EPIC Systems Verona, Wisconsin). The data was collected from August 2016 to April 2022. The following data were extracted from the EMR for the visit: age, sex, race, hospital LOS, systolic and diastolic blood pressure values during the ED visit, medications administered while in the ED, ICD-10 code diagnoses while in the ED and during subsequent hospital stay, hospital LOS, discharge disposition setting, and ICU admission status. Antihypertensive medications pulled from EPIC EMR were reviewed and encompassed drug classes such as direct renin inhibitors, calcium blockers, angiotensin-converting enzyme (ACE) inhibitors, angiotensin II receptor blockers (ARBs), as well as diuretics and alpha/beta-blockade with indications for hypertension. With regards to the diagnoses in the subsequent hospital stay, we evaluated the outcomes of ischemic and hemorrhagic stroke (ICD-10 I63.0-163.9, I62.9) and death. The sample size and power analysis were determined via a Pearson's Chi-squared test conducted by the statistician wherein power was set to 0.8, alpha to 0.05 and the test was set to two-sided. Our study protocol followed the medical record review criteria proposed by Worster et al., with the exception of three criteria that did not apply to our study (use of an abstraction form and interobserver reliability discussion or testing method) [16].

Statistical analysis

Descriptive statistics were used to summarize patient characteristics. Numerical variables were summarized using the median and interquartile ranges, whereas categorical variables were displayed using frequencies and percentages. Statistical analysis was performed as a method of detecting any differences between the antihypertensive treatment group and the no-treatment group. The comparison of numerical variables was done using t-tests, and to test the association between categorical variables, the Chi-square test was used. In order to determine the effect that antihypertensive therapy had on stroke rate, a univariable logistic regression model was performed, with antihypertensive treatment as the independent variable and stroke as the outcome variable. Further, a multivariable logistic regression model was run, again, with stroke as the outcome variable and antihypertensive treatment as a predictor variable, this time adjusting for age, sex, and race. The patient cohorts encompassing stage 1 and stage 2 hypertension, respectively, were also analyzed separately with both a univariable and multivariable logistic regression model using ischemic stroke as the outcome variable and antihypertensive treatment group as the predictor variable, with and without adjusting for age, sex, and race. For the purposes of this analysis, stage 1 hypertension was defined as having a systolic blood pressure of between 140 and 159 mm Hg or a diastolic blood pressure in the range of 90-99 mm Hg, whereas those with a diastolic or systolic blood pressure of 160 mm Hg or 100 mm Hg, respectively, any time during the duration of their ED stay were deemed to be in the stage 2 hypertensive group.

Subsequent secondary two-sample analyses with regards to ICU admission, discharge disposition setting, and hospital LOS, individually, were compared between groups of patients receiving antihypertensive medications and patients not receiving antihypertensive medications. To determine whether there was an association between ICU admissions and antihypertensive medications administered, a Chi-square test was used. A Fisher's exact test was used to test for an association between discharge disposition setting and antihypertensive medication administration in the ED. Finally, a t-test was used to test if there was a difference in mean LOS between the two groups. Analyses were done using the Statistical Analysis System (SAS) (v9.4; SAS Institute Inc., Cary, NC). Two-sided tests were used and alpha was set to 0.05. Missing values were listed when appropriate but have been excluded from statistical analysis.

## Results

Patient and clinical characteristics

A total of 3,417 adult patients were seen at the study hospital system’s EDs between August 2016 and April 2022 with a discharge diagnosis of TIA and a systolic blood pressure of 140 mm Hg or greater or a diastolic blood pressure of 90 mm Hg or greater. After applying exclusion criteria, 3,095 patients remained in the study cohort (Table 1). A total of 649 (21%) patients received an antihypertensive medication while in the ED whereas 2,446 (79%) did not. The median age of the entire cohort was 73.0 years, with the group receiving antihypertensive therapy being slightly older. The sample had more females (1726 (55.8%)) than males (1369 (44.2%)). Of the 1,726 females, 385 (22.3%) received an antihypertensive in the ED, and, of the 1,369 males, 264 (19.3%) received an antihypertensive in the ED (p = 0.0403) (Table 1). Regarding race, a majority of the patients identified as White individuals or Caucasian 2,322 (75.1%), 627 (20.3%) identified as Black individuals or African American, and 142 (4.6%) identified as Other. Those identifying as White individuals or Caucasian had the lowest proportion receiving antihypertensive medication when compared to both those identifying as Black individuals or African American and as Other (p < 0.0001) (Table 1). Of the 3,095 total patients, 1,074 (34.7%) were in the stage 1 hypertensive group, whereas 2,021 (65.3%) of the patients were in the stage 2 hypertension group. Those who had stage 2 hypertensive BP levels in the ED had a statistically significantly higher proportion receiving antihypertensive treatment in the ED (570 (28.2%)) when compared to stage 1 patients (79 (7.4%)) (p < 0.0001) (Table 1).

**Table 1:** Patient and clinical characteristics T-value^1^; Chi-square value^2^; Unequal variance two sample t-test p-value^3^; Chi-square p-value^4^ The data has been represented as n (%) aside from age where median (IQR) was used; Significance defined as p < 0.05. Stage 1 hypertension: SBP = 140-159 mm Hg or DBP = 90-99 mm Hg; Stage 2 hypertension: SBP ≥ 160 mm Hg or DBP ≥100 mm Hg SBP: systolic blood pressure; DBP: diastolic blood pressure; ED: emergency department

Variables		Antihypertensive administered in ED		Test value	p-value
	All	Yes	No		
n (%)	3095	649 (21)	2446 (79)		
Age, median (IQR)	73.0 (62.0, 82.0)	75.0 (65.0, 83.0)	73.0 (62.0, 82.0)	-4.4^1^	<0.0001^3^
Sex, n (%)				4.2^2^	0.0403^4^
Female	1726 (55.8)	385 (22.3)	1341 (77.7)		
Male	1369 (44.2)	264 (19.3)	1105 (80.7)		
Race, n (%)				23.7^2^	<0.0001^4^
White individual or Caucasian	2322 (75.1)	440 (18.9)	1882 (81.1)		
Black individual or African American	627 (20.3)	172 (27.4)	455 (72.6)		
Other	142 (4.6)	37 (26.1)	105 (73.9)		
Missing	4	0	4		
Stage of hypertension, n (%)				183.9^2^	<0.0001^4^
Stage 1	1074 (34.7)	79 (7.4)	995 (92.6)		
Stage 2	2021 (65.3)	570 (28.2)	1451 (71.8)		

Stroke outcomes between groups

Of the 3,095 patients in our study cohort, 429 patients (13.9%) had a stroke during their subsequent hospital stay with 419 (13.5%) of these strokes being ischemic and nine (0.29%) being hemorrhagic (Table 2). One patient developed both an ischemic and hemorrhagic stroke. Of those who developed an ischemic stroke, 95 patients (22.7%) received an antihypertensive in the ED whereas 324 (77.3%) did not (Table 2). We observed no significant difference in ischemic stroke rate in the treatment group compared to the no-treatment group after adjusting for age, sex, and race (adjusted odds ratio (aOR), 1.11 (95% confidence interval (CI), 0.87-1.43), p = 0.399) (Table 2). Of the 419 patients suffering from an ischemic stroke, 122 (29.1%) were in the stage 1 hypertensive group whereas 298 (71.1%) were in the stage 2 hypertensive group (Table 2). With regards to antihypertensive treatment in the ED within a given stage of hypertension, we observed no significant difference in stroke in the treatment group compared to the no-treatment group when adjusting for age, sex, and race in either the stage 1 hypertensive group or in stage 2 hypertensive group respectively (aOR, 0.79 (95% CI, 0.36-1.71), p = 0.548) (aOR, 1.05 (95% CI, 0.80-1.38), p = 0.731) (Table 2).

**Table 2:** Effect of administration of antihypertensive medication on stroke risk in ED patients presenting with TIA and elevated blood pressure Wald Chi-square^1^ Unadjusted odds ratio (OR) estimated by univariable regression. Adjusted odds ratio (OR) was estimated by multivariable logistic regression, adjusting for age, sex, and race. The data has been represented as n (%); Significance is defined as p < 0.05. Stage 1 hypertension: SBP = 140-159 mm Hg or DBP = 90-99 mm Hg; Stage 2 hypertension: SBP ≥ 160 mm Hg or DBP ≥100 mm Hg SBP: systolic blood pressure; DBP: diastolic blood pressure; ED: emergency department

Outcomes		Antihypertensive administered in ED		Unadjusted OR (yes vs no)	Test value^1^	p-value	Adjusted OR (yes vs no)	Test value^1^	p-value
	All	Yes	No						
n (%)	3095	649 (21)	2446 (79)						
Total stroke	429 (13.9)	98 (22.8)	331 (77.2)	1.14 (0.89-1.45)	1.05	0.304	1.12 (0.87-1.43)	0.80	0.371
Ischemic stroke only	419 (13.5)	95 (22.7)	324 (77.3)	1.12 (0.88-1.44)	0.85	0.357	1.11 (0.87-1.43)	0.71	0.399
Stage 1 hypertension	122 (29.1)	8 (6.6)	114 (93.4)	0.87 (0.41-1.86)	0.13	0.720	0.79 (0.36-1.71)	0.36	0.548
Stage 2 hypertension	297 (70.9)	87 (29.3)	210 (70.7)	1.06 (0.81-1.40)	0.20	0.652	1.05 (0.80-1.38)	0.12	0.731
Hemorrhagic stroke only	9 (0.29)	3 (33.3)	6 (66.7)	1.89 (0.47-7.57)	0.81	0.369			
Ischemic/hemorrhagic stroke	1 (0.03)	0 (0)	1 (100)						

Comparison of secondary outcomes between groups

We observed a small increase in hospital LOS in the antihypertensive treatment group compared to the no-treatment group (2.1 days vs 1.9 days) (p < 0.0001) (Table 3). No significant differences were seen in ICU admissions (2.8% vs 2.2%) (p = 0.359) or discharge disposition setting when it came to home discharge, rehab/intermediate care/skilled nursing facility, death, leaving against medical advice (AMA), or not applicable/other (p = 0.137) among those who received antihypertensive therapy in the ED and those who did not (Table 3).

**Table 3:** Effect of administration of antihypertensive medication on secondary outcomes in ED patients presenting with TIA and elevated blood pressure *Monte Carlo Estimate (20,000 samples); T-value^1^; Chi-square value^2^; Fisher Exact p-value^3^; Unequal variance two sample t-test p-value^4^; Chi-square p-value^5^ The data has been represented as n (%) aside from hospital length of stay where median (IQR) was used; Significance is defined as p < 0.05. ED: emergency department; AMA: against medical advice

Outcomes		Antihypertensive Administered in ED		Test value	p-value
	All	Yes	No		
Discharge disposition, n (%)				*	0.1371^3^
Home discharge	2695 (88.4)	548 (86.0)	2147 (89.0)		
Rehab/intermediate care/skilled nursing facility	299 (9.8)	76 (11.9)	223 (9.2)		
Deceased	3 (0.1)	1 (0.2)	2 (0.1)		
Left AMA/discontinued care	48 (1.6)	10 (1.6)	38 (1.6)		
Not applicable/other	5 (0.2)	2 (0.3)	3 (0.1)		
Missing	45	12	33		
Hospital length of stay (days), median (IQR)	2.0 (1.2, 3.1)	2.1 (1.5, 3.8)	1.9 (1.2, 3.0)	-4.9^1^	<0.0001^4^
Intensive care unit admission, n (%)				0.84^2^	0.3587^5^
Yes	71 (2.3)	18 (2.8)	53 (2.2)		
No	3024 (97.7)	631 (97.2)	2393 (97.8)		

## Discussion

Hypertension is implicated as a major risk factor for the development of a TIA and in the incidence of a subsequent stroke following a TIA [5,12]. In this observational study, we investigated the outcomes of ED antihypertensive therapy in TIA patients with elevated blood pressure in the ED. In this population of TIA patients, we observed no difference in stroke frequency during the subsequent hospital stay between individuals treated with an antihypertensive medication and those who were not. Furthermore, no relationship was observed between the degree of hypertension and antihypertensive administration in the ED on ischemic stroke outcomes. In addition to including a highly diverse patient population, this study is, to our knowledge, one of the largest retrospective reviews investigating the outcomes of acute antihypertensive therapy in TIA patients presenting to the ED with elevated blood pressure.

There is an abundance of research demonstrating the efficacy of long-term blood pressure control for stroke prevention in patients with a history of TIA [17]. Accordingly, the American Heart Association recommends starting antihypertensive treatment within several days of a TIA in patients with established blood pressures of ≥ 140/90 mm Hg with the goal of reducing stroke risk [13]. However, the optimal timing of initiation of antihypertensive therapy following a TIA is unclear, and to our knowledge, this is the first study to investigate stroke risk after initiating antihypertensive treatment in the ED.

Our results indicate that in ED patients presenting with TIA and elevated blood pressure, ED antihypertensive therapy is not associated with decreased stroke rates during the subsequent hospital admission. Stroke and TIA are complex conditions with multiple risk factors and etiologies, and it appears that antihypertensive treatment alone may not be enough of an intervention to have a clinically meaningful effect. The ABCD^2^ risk score, which estimates stroke risk after a TIA, encompasses not only high blood pressure, but also advanced age, diabetes history, TIA duration, and patient symptomatology, again pointing to the multifactorial nature of stroke [8,9]. Furthermore, as ischemic stroke typically results from arterial hypoperfusion, acutely treating blood pressure in the setting of TIA, even if elevated, may result in worsening ischemia in some patients.

We also investigated the effects of antihypertensive treatment on subsequent hospital LOS, ICU admission, and discharge disposition settings. We observed an average hospital LOS of two days, consistent with prior research [18]. Administration of an antihypertensive in the ED was associated with minimally increased hospital LOS, but this is likely of no clinical significance due to the minimal duration of the difference. No difference was observed between the treatment and no treatment groups in the ICU admit rate or discharge disposition setting. Given the transient effect of an ED antihypertensive treatment, the lack of downstream effects on hospital LOS, ICU admission rates, and discharge disposition settings is not surprising.

Limitations

This study had several limitations. First, this is a retrospective study, and, therefore, no causal relationship can be established between antihypertensive treatment and outcomes in this patient cohort. Second, we relied upon physician documentation of TIA which has varying presentations and no confirmatory testing, so it is likely that we missed patients that had true TIAs and possibly that patients in our population had TIA mimics. Third, the data pulled from the EMR was only from a single healthcare system, however, it does encompass a diverse socioeconomic population. Finally, given the retrospective nature of this investigation, it is unclear if the antihypertensive was given with the intention of lowering stroke risk or for a different purpose. Future studies should prospectively investigate the role of antihypertensive management acutely in the setting of TIA in the ED. Additionally, future studies should look into the role of specific antihypertensive classes and their effect on stroke outcomes.

## Conclusions

In this retrospective study, we investigated the outcomes of ED antihypertensive therapy in TIA patients with elevated blood pressure in the ED. In this cohort of TIA patients with elevated blood pressure in the ED, there was no observed subsequent difference in stroke risk between patients who received antihypertensive therapy compared to those who did not. As there does not appear to be any statistically significant effect on stroke risk in the subsequent hospital stay, ED management of elevated BP in TIA patients should be guided by physician discretion and clinical experience.
